# Serum Malondialdehyde Levels in Patients with Malignant Middle Cerebral Artery Infarction Are Associated with Mortality

**DOI:** 10.1371/journal.pone.0125893

**Published:** 2015-05-01

**Authors:** Leonardo Lorente, María M. Martín, Pedro Abreu-González, Luis Ramos, Mónica Argueso, Jordi Solé-Violán, Marta Riaño-Ruiz, Alejandro Jiménez

**Affiliations:** 1 Intensive Care Unit, Hospital Universitario de Canarias, Santa Cruz de Tenerife, Spain; 2 Intensive Care Unit, Hospital Universitario Nuestra Señora de Candelaria, Santa Cruz de Tenerife, Spain; 3 Deparment of Phisiology, Faculty of Medicine, University of the La Laguna, La Laguna. Santa Cruz de Tenerife, Spain; 4 Intensive Care Unit, Hospital General La Palma, Breña Alta, La Palma, Spain; 5 Intensive Care Unit, Hospital Clínico Universitario de Valencia, Valencia, Spain; 6 Intensive Care Unit, Hospital Universitario Dr. Negrín, CIBERES, Las Palmas de Gran Canaria, Spain; 7 Servicio de Bioquímica Clínica, Complejo Hospitalario Universitario Insular Materno-Infantil, Las Palmas de Gran Canaria, Spain; 8 Research Unit, Hospital Universitario de Canarias, La Laguna, Santa Cruz de Tenerife, Spain; University of Leicester, UNITED KINGDOM

## Abstract

**Objective:**

Malondialdehyde (MDA) is an end-product formed during lipid peroxidation, due to degradation of cellular membrane phospholipids. MDA is released into extracellular space and finally into the blood; it has been used as an effective biomarker of lipid oxidation. High circulating levels of MDA have been previously described in patients with ischemic stoke than in controls, and an association between circulating MDA levels and neurological functional outcome in patients with ischemic stoke. However, an association between serum MDA levels and mortality in patients with ischemic stroke has not been previously reported, and that was the objective of this study.

**Methods:**

Observational, prospective and multicenter study performed in six Intensive Care Units. We included patients with severe malignant middle cerebral artery infarction (MMCAI) defined as Glasgow Coma Scale (GCS) lower than 9. We measured serum MDA levels in 50 patients with severe MMCAI at the time of diagnosis and in 100 healthy subjects. Mortality at 30 days was the end point of the study.

**Results:**

We found that patients with severe MMCAI showed higher serum MDA levels than healthy subjects (p<0.001). We found higher serum MDA levels (p<0.001) in non-surviving MMCAI patients (n=26) than in survivors (n=24). The area under the curve for prediction of 30-day mortality for serum MDA levels was 0.77 (95% CI = 0.63-0.88; p<0.001). Serum MDA levels >2.27 nmol/mL were associated with 30-day mortality (OR=7.23; 95% CI=1.84-28.73; p=0.005) controlling for GCS and age on multiple binomial logistic regression analysis.

**Conclusions:**

To our knowledge, this is the first study showing that serum malondialdehyde levels in patients with MMCAI are associated with early mortality.

## Introduction

Ischemic stroke is an important cause of disability, mortality and resource consumption [1]. Oxidative stress has been defined as a disturbance in the pro-oxidant and antioxidant balance in favour of the former, leading to potential damage [2]. Malondialdehyde (MDA) is an end-product formed during lipid peroxidation, due to degradation of cellular membrane phospholipids [3,4]. MDA is released into extracellular space and finally into the blood; it has been used as an effective biomarker of lipid oxidation [3,4].

Previous studies have found higher circulating levels of MDA in patients with ischemic stoke than in controls [5–17], and an association between circulating MDA levels and neurological functional outcome in patients with ischemic stroke [18–20].

We hypothesized that higher serum MDA levels in patients with ischemic stroke could be associated with early mortality due to the fact that in ischemic stroke exit lipid peroxidation, MDA is an effective biomarker of lipid oxidation, and higher circulating MDA levels have been associated with poor neurological functional outcome in ischemic stroke patients. However, an association between serum MDA levels and mortality in patients with ischemic stroke has not been reported. Therefore, the objective of this study was to determine whether there is an association between serum MDA levels and early mortality in patients with ischemic stroke.

## Methods

We included 50 patients with severe malignant middle cerebral artery infarction (MMCAI) and 100 healthy volunteer control subjects matched according to age and sex. The diagnosis of ischemic stroke was based on clinical and computed tomography findings [1]. The severity of MMCAI was classified according to Glasgow Coma Scale (GCS) [21], and severe was defined as a GCS ≤8. Patients aged less than 18 years, and those with pregnancy, inflammatory or malignant disease were excluded from the study.

A prospective, observational, multicenter study was performed in 6 Intensive Care Units between 2009–2012. The study was approved by the Institutional Review Board of all participating hospitals: Hospital Clínico Universitario de Valencia (Valencia, Spain), Hospital General de La Palma (La Palma, Spain), Hospital Universitario Dr. Negrín (Las Palmas de Gran Canaria, Spain), Hospital Insular (Las Palmas de Gran Canaria, Spain), Hospital Universitario Nuestra Señora de Candelaria (Santa Cruz de Tenerife, Spain), Hospital Universitario de Canarias (La Laguna, Santa Cruz de Tenerife, Spain). Written informed consent was obtained from the legal guardians of the patients.

We collected blood samples from 50 patients with severe MMCAI at the time of diagnosis (within 4 hours after diagnosis), and from100 controls to measure serum MDA concentrations. As we described previously [22], serum MDA levels were measured using a thiobarbituric acid-reactive substance (TBARS) method suggested by Kikugawa et al with some modifications [23].

We used SPSS 17.0 (SPSS Inc., Chicago, IL, USA) to perform the statistical analyses. We reported categorical variables as frequencies and percentages, and chi-square test was used for the comparisons between groups. We found that some continuous variables did not present a normal distribution according to Kolmogorov-Smirnov test; therefore, we used a non-parametric test for the comparison between groups. We reported continuous variables as medians and interquartile ranges, and Mann-Whitney U-test was used for the comparisons between groups. To determine the independent contribution of serum MDA levels on 30-day mortality, controlling for GCS and age, multiple binomial logistic regression analysis was used. To determine the goodness-of-fit of serum MDA levels for the prediction of 30-day mortality, receiver operating characteristic (ROC) analysis was used. We carried out a 30-day survival analysis using serum MDA levels lower or higher than 2.27 nmol/mL. We used Kaplan-Meier method for survival curves, and the survival time between both curves was compared using log-rank test. We used the maximum likelihood ratio between sensitivity and specificity to select the cut-off point for MDA levels. We considered statistically significant all P values lower than 0.05.

## Results

Table 1 shows age, sex and serum MDA levels of healthy subjects and patients with severe MMCAI.

**Table 1:** Characteristics of healthy controls and patients with severe malignant middle cerebral artery infarction.

	Healthy controls (n = 100)	Patients (n = 50)	p-value
Gender female—n (%)	38 (38.0)	17 (34.0)	0.72
Age—median years (p 25–75)	59 (47–71)	60 (51–69)	0.72
MDA—median nmol/mL(p 25–75)	1.11 (0.79–1.51)	2.16 (1.36–2.97)	<0.001

**Fig 1:**
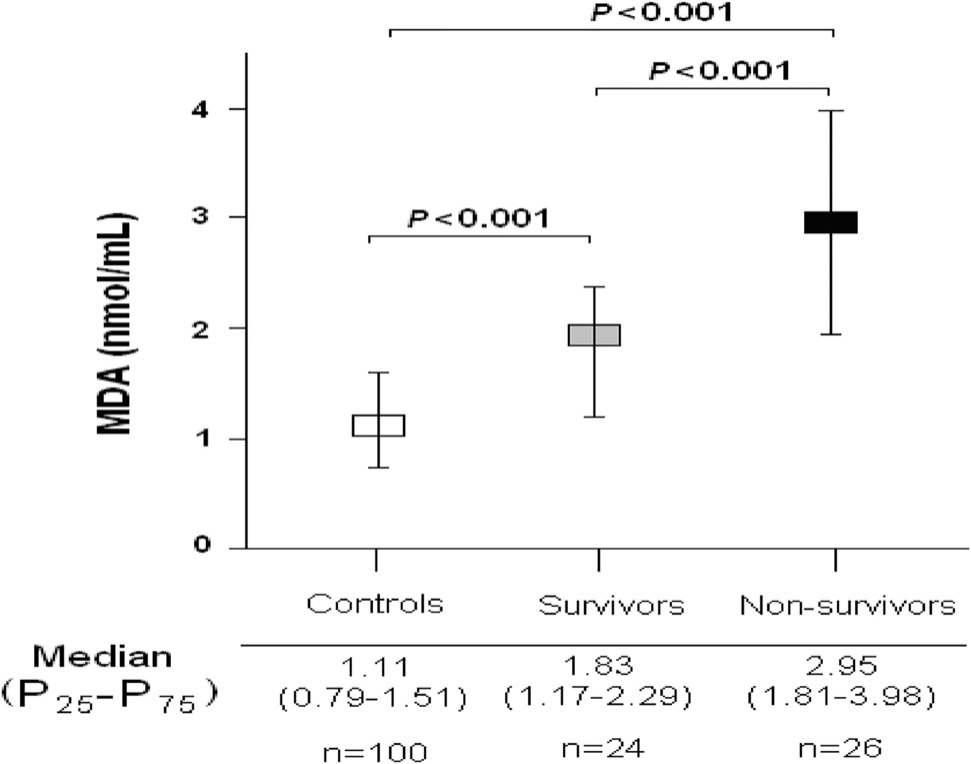
Serum malondialdehyde levels in severe malignant middle cerebral artery infarction patients and healthy controls.

We found that patients with severe MMCAI showed higher serum MDA levels (p<0.001) than healthy subjects (Fig 1).

Table 2 shows that non-surviving MMCAI patients (n = 26) had a tendency to higher age and creatinine levels and lower GCS than survivors (n = 24), and that non-surviving MMCAI patients had significantly lower platelet count and higher serum MDA levels (p<0.001) than survivors (Fig 1). Thrombectomy was not used in none patients.

**Table 2:** Clinical and biochemical characteristics of 30-day survivor and non-survivor MMCAI patients.

	Survivors (n = 24)	Non-survivors (n = 26)	P value
Age (years)—median (p 25–75)	47 (32–67)	66 (45–76)	0.14
APACHE-II score—median (p 25–75)	20 (16–25)	22 (19–29)	0.14
aPTT (seconds)—median (p 25–75)	28 (25–29)	26 (25–33)	0.96
Bilirubin (mg/dl)—median (p 25–75)	0.50 (0.38–0.90)	0.53 (0.30–1.20)	0.76
Creatinine (mg/dl)—median (p 25–75)	0.80 (0.60–1.10)	1.01 (0.85–1.45)	0.052
Decompressive craniectomy—n (%)	7 (29.2)	5 (19.2)	0.51
Fibrinogen (mg/dl)—median (p 25–75)	440 (335–494)	409 (322–598)	0.71
Gender female—n (%)	8 (33.3)	9 (34.6)	0.99
GCS score—median (p 25–75)	7 (6–8)	6 (4–8)	0.10
Glycemia (g/dL)—median (p 25–75)	133 (105–170)	135 (110–154)	0.92
Hemoglobin (g/dL)—median (p 25–75)	12.0 (11.3–13.8)	12.0 (11.0–15.1)	0.92
INR—median (p 25–75)	1.07 (1.01–1.20)	1.20 (1.07–1.48)	0.16
Lactic acid (mmol/L)-median (p 25–75)	1.25 (0.93–1.68)	1.50 (1.01–3.15)	0.08
Leukocytes-median*10^3^/mm^3^ (p 25–75)	12.8 (9.8–16.9)	14.4 (11.9–21.9)	0.49
MDA (nmol/mL)—median (p 25–75)	1.83 (1.17–2.29)	2.95 (1.81–3.98)	<0.001
PaO2 (mmHg)—median (p 25–75)	110 (101–194)	104 (85–139)	0.10
PaO2/FI0_2_ ratio—median (p 25–75)	246 (192–327)	248 (175–320)	0.41
Platelets—median*10^3^/mm^3^ (p 25–75)	227(183–308)	152 (123–190)	0.003
Sodium (mEq/L)- median (p 25–75)	140 (138–145)	140 (137–146)	0.91
Temperature (°C)—median (p 25–75)	36.5 (35.7–37.0)	37.0 (35.7–37.8)	0.26
Thrombolysis	5 (20.8)	8 (30.8%)	0.53

P 25–75 = percentile 25^th^-75^th^; APACHE II = Acute Physiology and Chronic Health Evaluation; aPTT = activated partial thromboplastin time; GCS = Glasgow Coma Scale; INR = international normalized ratio; PaO_2_ = pressure of arterial oxygen/fraction inspired oxygen; FIO_2_ = pressure of arterial oxygen/fraction inspired oxygen; MDA = Malondialdehyde

**Fig 2:**
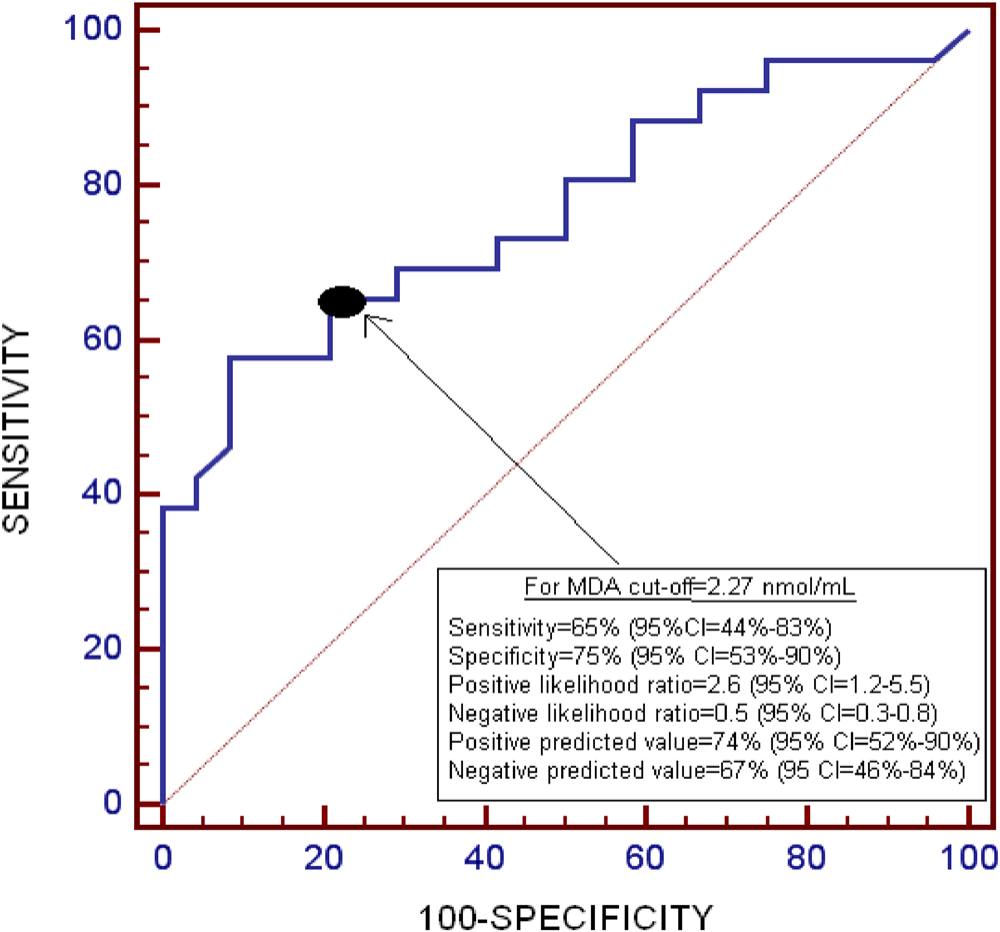
Receiver operation characteristic analysis using serum malondialdehyde levels as predictor of mortality at 30 days.

The area under the curve for the prediction of 30-day mortality for serum MDA levels was 0.77 (95% CI = 0.63–0.88; p<0.001) (Fig 2).

Serum MDA levels >2.27 nmol/mL were associated with 30-day mortality (Odds Ratio = 7.23; 95% CI = 1.84–28.73; p = 0.005) after controlling for GCS and age in the multiple binomial logistic regression analysis (Table 3).

**Table 3:** Multiple binomial logistic regression analysis to predict 30-day mortality.

Variable	Odds Ratio	95% Confidence Interval	*P*
Serum malondialdehyde levels>2.27 nmol/mL	7.23	1.84–28.73	0.005
Glasgow Coma Scale	0.70	0.49–1.01	0.06
Age (years)	1.03	0.98–1.09	0.23

**Fig 3:**
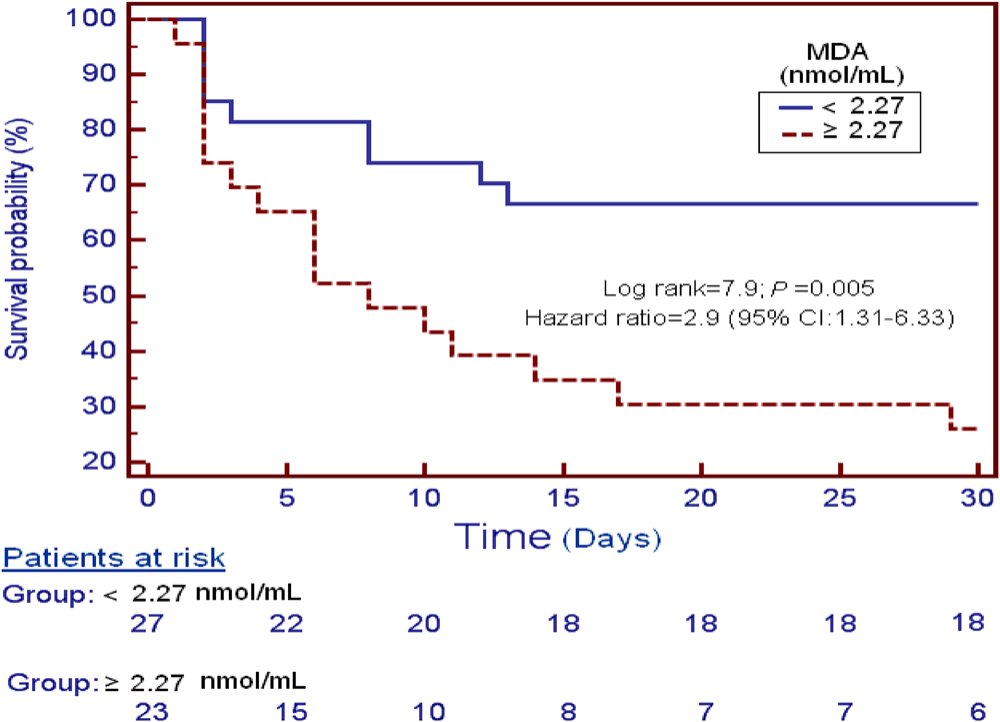
Survival curves at 30 days using serum malondialdehyde levels higher or lower than 2.27 nmol/mL.

Patients with serum MDA higher than 2.27 nmol/mL (n = 23) presented higher 30-day mortality (Hazard ratio = 2.9; 95% CI = 1.31–6.33; p = 0.005) than patients with lower levels (n = 27) in the survival analysis (Fig 3).

The causes of death were the following: brain death 36%, cardiac arrest secondary to intracranial hypertension 32%, and limitation of therapeutic effort 32%.

## Discussion

The novel findings of our study were: a) Higher serum MDA levels were observed in non-surviving than in surviving severe MMCAI patients; b) Serum MDA levels were associated with 30-day mortality in severe MMCAI patients; c) Serum MDA levels could be used as a prognostic biomarker in patients with severe MMCAI.

Higher circulating MDA levels have been reported in patients with ischemic stroke than in controls [5–17], and the present study showed similar results.

In addition, higher serum MDA levels were observed in non-surviving severe MMCAI patients than in survivors, which have not been previously reported. We also found an association between serum MDA levels and early mortality on logistic regression analysis, and that serum MDA levels could be used to predict early mortality according to ROC analysis.

We found that non-surviving MMCAI patients had a tendency to higher creatinine and significantly lower platelet count than survivors at the time of severe MMCAI diagnosis. It could be thought that the higher MDA levels in non-surviving patients than in survivors were due to secondary complications (such as pneumonia); however, we believe that lipid peroxidation is due to MMCAI, and more rarely due to secondary complications, because all variables were collected early.

In the multiple binomial logistic regression analysis to predict 30-day mortality, we included GCS and age since those variables have been associated with a poor prognosis in ischemic stroke patients [24].

We did not use the National Institutes of Health Stroke Scale (NIHSS) [25] to assess stroke severity because all the patients in our study were in coma (GCS ≤8), which made it very difficult to evaluate the different items of this scale; and for this reason we used GCS to assess stroke severity.

The findings are in consonance with the results of previous studies showing higher MDA levels in ischemic stroke patients with poor functional outcome [18–20], and with the findings of other studies reporting an association between circulating MDA levels and mortality in patients with brain trauma injury [22] and sepsis [26]. We believe that MDA level might be a predictor of outcome after ischemic stroke, traumatic brain injury and sepsis since MDA is an effective biomarker of lipid oxidation [3,4], and in all these clinical situations oxidative stress and lipid peroxidation are present.

Taken togheter, our findings suggest that an alteration of the oxidative state may be of great pathophysiological significance in patients with MMCAI. The higher circulation levels of MDA found in patients with MMCAI than in controls and in non-survivors than in surviving MMCAI patients, represents higher lipid peroxidation due to overproduction of ROS and RNS caused by the imbalance between antioxidant and pro-oxidant systems.

The administration of antioxidant agents could have a beneficial effect in MMCAI patients. In some clinical trials, acute ischemic stroke patients were randomly assigned to receiving different antioxidant vitamins (E, C, B2, B6, B12) or not [27–29]. A decrease in plasma MDA concentration was observed in the treatment group compared with the control group.

Our study has certain limitations. First, the determination of other compounds of oxidant state could be interesting. Second, we did not report data about the evolution of serum MDA levels in non-surviving and surviving MMCAI patients. Third, the timing of the blood sampling may have changed among patients. Fourth, the inclusion of very severe stroke patients may limit the generalisability of the results to other ischemic stroke patients. Fifth, the lesion volume on computed tomography to assess the extent of damaged tissue volume has not been reported. Sixth, we have not reported data about the risk factor profile in patient and control populations; however, the objective of this study was to determine whether there is an association between serum MDA levels and mortality in patients with ischemic stroke. Finally, the sample size of the study was not pre-determined and was relatively small; however, our non-probabilistic sample was large enough to be able to show an association between serum MDA levels and mortality in patients with MMCAI. The main implications of our study findings are that serum MDA levels could be used to predict outcome in patients with severe MMCAI and could generate interest for research into the use of antioxidant agents in patients with severe MMCAI; however, additional studies are therefore needed to confirm the main finding of our study.

## Conclusions

To our knowledge, this is the first study showing that serum malondialdehyde levels in patients with MMCAI are associated with early mortality.
